# Regional variations distinguish auditory from visual evoked potentials in healthy 4 week old piglets

**DOI:** 10.1088/1361-6579/acb4da

**Published:** 2023-02-28

**Authors:** R Anna Oeur, Maduran Palaniswamy, Matthew Ha, Mariano Fernandez-Corazza, Susan S Margulies

**Affiliations:** 1 Wallace H. Coulter Department of Biomedical Engineering, Emory University and Georgia Institute of Technology, Atlanta, Georgia, United States of America; 2 LEICI Instituto de Investigaciones en Electrónica, Control y Procesamiento de Señales, Facultad de Ingeniería, Universidad Nacional de La Plata, Buenos Aires, Argentina

**Keywords:** auditory oddball paradigm, EEG, porcine, visual event-related potentials, source localization

## Abstract

*Objective.* Evoked potentials (EP), measured using electroencephalographic (EEG) recordings provide an opportunity to monitor cognitive dysfunctions after neurological diseases or traumatic brain injury (TBI). The 4 week old piglet is an established model of paediatric TBI; therefore, healthy piglets were studied to establish feasibility of obtaining responses to auditory and visual stimuli. A secondary aim was to input the EEG data into a piglet computational model to localize the brain sources related to processing. We tested the hypotheses: (1) visual, auditory-standard, and auditory-target stimuli elicit responses, (2) there is an effect of stimulus type, day tested, and electrode region on EPs from EEG, (3) there is an effect of stimulus type, day tested, and brain region on localized sources from a computational model. *Approach.* Eleven 4 week old female piglets were fitted with a 32-electrode net and presented with a simple white light stimulus and an auditory oddball click train (70 standard; 30 target tones). *Main results.* *N*1 and *P*2 amplitudes were consistently observed for all stimulus types. Significant interaction effects between brain region and stimulus for EP and current density demonstrate that cognitive responses are specific to each modality with auditory localizing to the temporal region and visual to the occipital regions. There was a day effect where larger responses were found on the first day than day 2 and 3 and may be due to the novelty of the stimulus on the first day. Visual stimuli had larger *P*1 amplitudes and earlier latencies (*P*1, *N*1) than auditory which coincides with current density results at 50 ms where larger activations were observed for visual. At 85 ms, auditory had significantly larger current densities coincident with larger and longer *N*1 amplitudes and latencies than visual. *Significance.* Auditory and visual processing were successfully and consistently obtained in a porcine model and can be evaluated as a diagnostic assessment for TBI.

## Introduction

Electroencephalography (EEG), in particular evoked potentials (EP) play an important role in detecting conduction abnormalities in neurological diseases and assessing central nervous system (CNS) pathways as the integrity of neurons are often affected (Lascano *et al* 2017). Different types of stimuli elicit different cortical responses in the brain and can provide a global assessment of the brain and the effects of the disease on normal functioning. Visual and auditory EPs have been used as diagnostic indicators of neurological diseases. Visual EPs assess the integrity of the visual pathway from the retina to the occipital lobe and provides an indicator of the health of the optic nerve (Lascano *et al* 2017). Auditory EPs assess the auditory pathway from the cochlea to the auditory cortex (Washnik *et al* 2019). After an auditory or visual stimulus, a series of local amplitude (uV) minima and maxima can be observed in the encephalographic waveform, and are generally termed *P*1 (50–90 ms), *N*1 (75–150 ms), *P*2 (159–230 ms), and *N*2 (150–250 ms). In the human subject, *P*1 is associated with sensory perception, *N*1 is related to sensory processing and stimulus discrimination, *P*2 and *N*2 have been associated with late-stage processing such as object recognition or categorization (Woodman 2010, Washnik *et al* 2019). Abnormalities and processing deficits as reflected by delayed or attenuated visual and auditory EPs have been identified in patients with Alzheimer’s disease (Tachibana *et al* 1996), Parkinson’s disease (Güntekin *et al* 2020), mild traumatic brain injury (Moore *et al* 2015), and multiple sclerosis (Covey *et al* 2021). In humans, Alzheimer’s disease is characterized by plaques, neurofibrillary tangles, and synaptic loss which results in memory loss and cognitive impairment. Multiple sclerosis is a disease where the immune system attacks the myelin covering of neurons in the brain and spinal cord causing decreased coordination, vision loss, and cognitive impairment. Parkinson’s disease is caused by a breaking down of neurons that produce dopamine, a neurotransmitter necessary for movement, normal brain activity, and speech. Traumatic brain injury (TBI) is a result of a trauma to the CNS that causes alterations in cognition and consciousness and can be marked by brain lesions, axonal pathologies, or transient functional deficits. While each of these diseases and injuries are marked by unique pathological and neurofunctional markers, a common denominator in each disease is abnormal brain activity.

Pre-clinical models of human neurological diseases are an important tool in advancing the science and treatment methods to mitigating and curing diseases (Chesselet and Carmichael 2012). Swine are a valuable large animal model for neuroscience, particularly for modelling the human brain (Duhaime 2006, Lind *et al* 2007). Swine have similar brain development trajectories as humans, from infant, toddler, adolescent ages (Ryan *et al* 2018a, 2018b), which include well circumscribed gyri and sulci patterns, and grey and white matter tissue distributions. These neuroanatomical characteristics are important factors for neuroimaging and eliciting EP that are easily measured on the scalp (Lind *et al* 2007) as well as replicating the distribution of axonal pathologies reported in human TBI (Smith *et al* 1999). Swine models have been developed for Alzheimer’s disease (Kragh *et al* 2009), multiple sclerosis (Singer *et al* 2000), Parkinson’s disease (Mikkelsen *et al* 1999), and traumatic brain injury (Margulies *et al* 2015). Brainstem auditory and visual EP were studied in juvenile and adult Vietnamese mini-potbellied pigs for the purpose of establishing baseline levels for neurological diseases (Strain *et al* 2006). The authors found an effect of age and maturation in EP parameters noting younger pigs having decreased amplitudes and longer latencies in comparison to adult pigs cautioning applying adult normative data to a younger population. In an effort to establish a swine model of schizophrenia, Arnfred *et al*, investigated auditory EP in healthy adult Gottingen minipigs using an auditory gating and auditory oddball paradigm (Arnfred *et al* 2003, 2004). From these studies, the authors concluded that sound processing is initiated in the auditory cortex below the sylvian fissure and further processing of tones have a frontal-global distribution. Understanding the distinctive regional brain functional and structural neuropathologic responses, and their interdependence are critical to identify the etiology of neurological diseases and TBI as well as strategies for brain injury prevention (Arnfred *et al* 2003, Margulies *et al* 2015). Pigs are an excellent model to study TBI because factors affecting the mechanics and trauma loads can be controlled, such as velocity and direction of head and brain motion (Cullen *et al* 2016). Often in clinical studies, diverse mechanisms of trauma as well as poly-traumas can cause injury to the brain in a non-uniform manner across subjects, resulting in a heterogenous study group contributing to non-significant results (Gomes and Damborská 2017). Additionally, swine are an important large animal model to develop clinical assessments employing visual and auditory EPs as these tools are non-invasive, require non-verbal responses, are objective and passive, thereby increasing translation from the pre-clinical animal to the human. The aim of this study was to establish auditory and visual EPs as a diagnostic tool for neurological assessment. A second aim of this study is to localize the EEG sources in the brain related to auditory and visual processing using geometrically realistic piglet head models to enhance our understanding of the local amplitude and regional effects of electrical activity while also serving as a proof of concept of a piglet model for source localization. Therefore, we first studied visual and auditory EPs in healthy piglets and tested the following hypotheses: (1) That visual stimuli would elicit responses unique to the regions associated with visual processing (occipital cortex) and auditory-standard, and auditory-target stimuli elicit responses specific to the auditory processing (auditory cortex) in piglets (2) There is an effect of stimulus type, day tested, and electrode region on EP from EEG (3) There is an effect of stimulus type, day tested, and brain region of localized sources determined from a computational model. To our knowledge this is a first study that captures visual and auditory EPs in healthy piglets and applies this study to a piglet brain model to localize sources.

## Methods

Eleven healthy 4 week old female Yorkshire piglets (*Sus scrofa*) were used in this study (Palmetto Research Swine, Reevesville, SC and Oak Hill Genetics, Ewing, IL). Emory University’s Institutional Animal Care and Use Committee (IACUC) approved all procedures. Piglets were socially housed in a cage and were on a 7 am–7 pm day–night cycle. Water and food were provided *ad libitum* (LabDiet 5080, St. Louis, MO). Animals were acclimated to EEG headgear and a sling for at least two 30 min sessions *prior* to testing. On test day, animals were placed in the sling, tended by two research staff, and a custom 32-electrode piglet net (EGI, Eugene, OR), that was soaked in a potassium chloride, water, and baby shampoo solution, was placed on the head. A light nylon stocking secured the electrode net to ensure scalp contact during testing. Electrical impedance for all electrodes was kept below 1 kΩ *prior* to testing.

### Stimulus presentation

**Figure 1.**
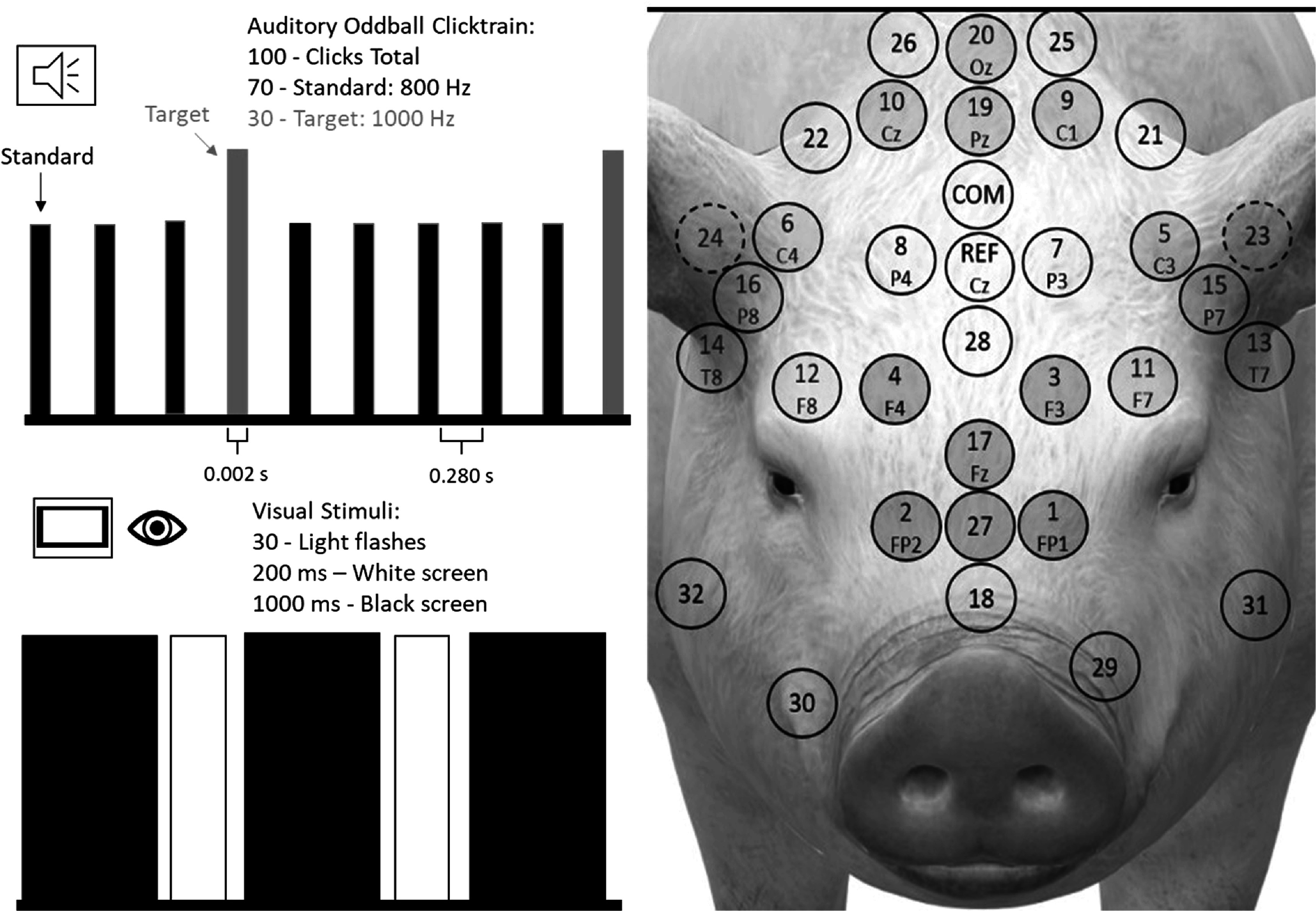
Illustration of auditory oddball paradigm in which 70 standard-800 Hz tones are played amidst 30 target-1000 Hz tones in random order, and visual stimulus sequence where a white computer screen is alternated with a black screen (left). The position of 32-electrode net on piglet head (right) and electrode groups are highlighted for front (red), temporal right (yellow), temporal left (green) and occipital (blue).

The visual trial consisted of 30 presentations of a white square on a black background (200 ms) followed by a black screen (1000 ms) on a portable 7’ LCD monitor held approximately 20 cm from the left eye of the piglet (107 lux). The auditory trial was an oddball click train of 100 tones played on a speaker (75 dBA) approximately 50 cm from the centre of the piglet’s head (figure 1), composed of 30 target tones (1000 Hz) presented randomly amidst 70 standard tones (800 Hz). Each tone lasted 2 ms and separated by 280 ms intervals. On each test day, each animal was presented with six visual trials and six auditory trials. The order of the tests (auditory first or visual first) was decided based on the subject’s temperament and whether the animal’s eyes remained open or closed when first placed in the sling. If the animal’s eyes were opened then visual trials were conducted first and vice versa. Animals were given breaks and food rewards in between each visual or auditory trial as needed. The Institutional Animal Care and Use at Emory University approved all procedures.

### Data acquisition and analysis

**Figure 2.**
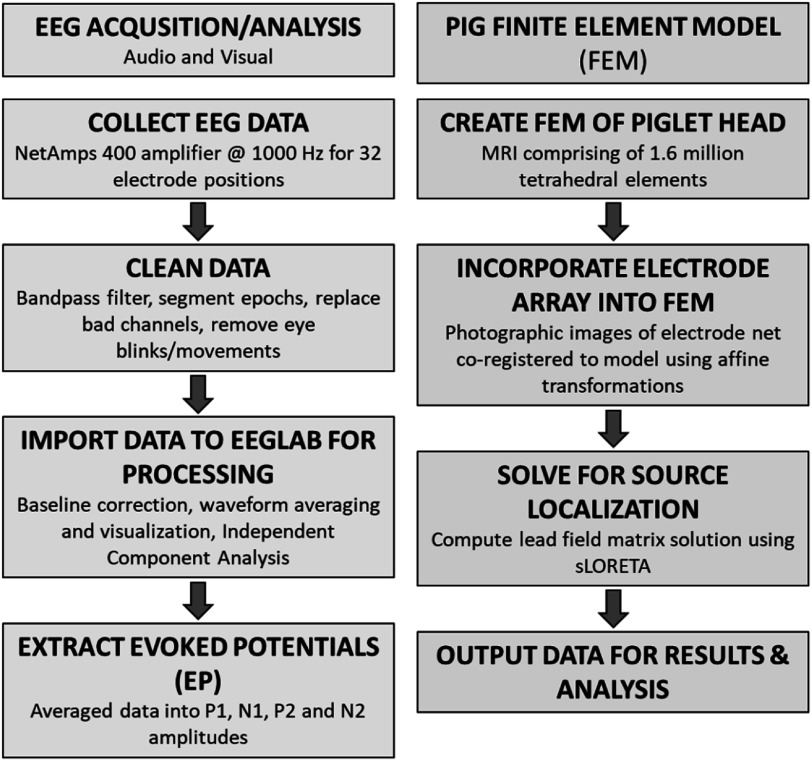
Flow charts for data acquisition and processing for EEG (left) and finite element model (FEM) development and source localization analysis (right).

A NetAmps 400 amplifier and Macbook Pro laptop with Netstation Acquisition software (Ver. 5.0) collected EEG data at 1000 Hz. Both visual and auditory stimuli were presented using a PC desktop computer with E-Prime software (Ver. 2.0, Psychology Software Tools Inc., Pittsburgh, PA). Netstation Tools (EGI, Eugene, OR) was used to apply a bandpass filter (0.1–30 Hz) to the data, segment epochs (50 ms before and 250 ms after each stimuli), replace bad channels (>200 uV), remove eye blinks (>140 uV) and eye movements (>55 uV). Data were then imported into EEGlab (V.14.12) and Matlab (V. R2019a, The Mathworks Inc., Natick, MA) for additionally processing. These steps included baseline correction, waveform averaging and visualization, and independent component analysis to isolate synchronized brain activity from spontaneous muscle artefact and eye movement (Makeig and Onton 2012). Waveforms in response to auditory-standard, auditory-target, and visual stimuli were averaged for each animal per day and peak amplitudes and latencies pertaining to the *P*1, *N*1, *P*2 and subsequent *N*2 attributes of the evoked potential were extracted (figure 2). Amplitude and latency data were then taken from six electrodes (1, 2, 3, 4, 17 and 27) covering the front of the head, five electrodes for the left (5, 11, 13, 15, 23) and right sides (6, 12, 14, 16, 24), and four electrodes covering the occipital (9, 10, 19, 20) region (figure 1) and were averaged per attribute to represent a single data point at each region per animal, per day, per stimulus type. Electrode regions defined here are used for analysis of variances further described below.

### Piglet finite element model

**Figure 3.**
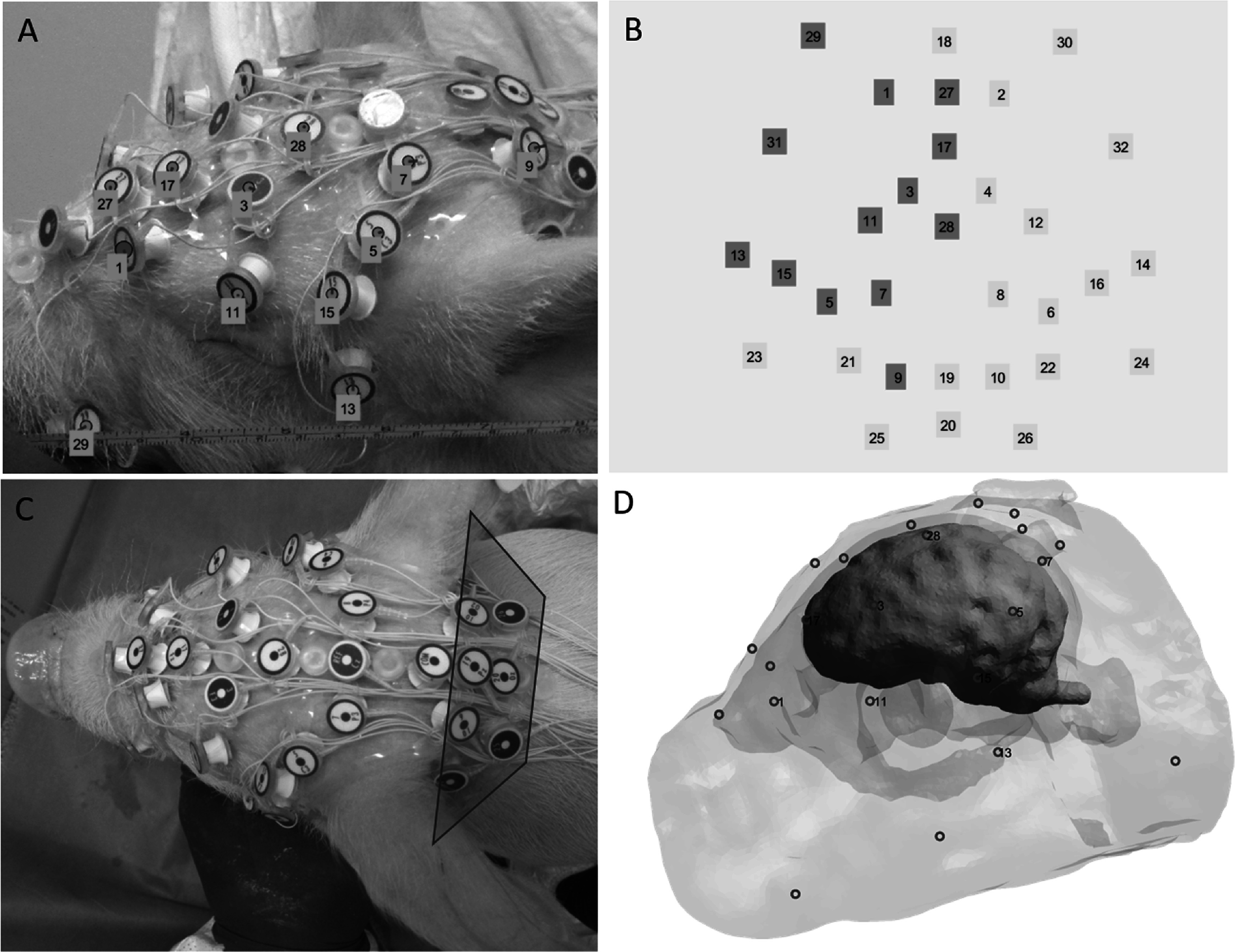
Images illustrating (A) electrode identification on the piglet head and (B) importing the locations into the ElectrodeWizzard platform. (C) Electrodes 9, 10, 19, 20, 21, 22, 25, 26 were not included in the model and are shown to be behind the ears. (D) Image of the piglet brain model with electrodes superimposed on the scalp surface.

To examine patterns of brain electrical activity associated with visual and auditory processing in the 4 week old piglet, EEG data were input into a finite element model (FEM) of the piglet head and brain to estimate the cortical current density distributions (figure 2). The piglet brain model used in this study is a scaled version of an infant piglet model originally developed for studying intracranial haemorrhage using electrical impedance tomography (Fernandez-Corazza *et al* 2016). The geometry of the finite element model was derived from magnetic resonance images (MRI) of a piglet and was segmented into the scalp, skull, brain, ventricles, and eyes, comprising 1.6 million tetrahedral elements. The segmentation was done slice by slice in a semi-automatic manner using the contour tool of the MIPAV software (https://mipav.cit.nih.gov/). The iso2mesh package (Fang and Boas 2009) was used to generate the triangular surfaces from the segmented masks, to smooth and merge them and to generate the tetrahedral mesh. The original model was scaled to the size of the 4 week old piglet brain and modified to incorporate the 32-electrode array. In order to accomplish this, a series of photographic images were taken of a 4 week old piglet fitted with the 32-electrode net in the coronal, horizontal, and sagittal planes, whereby each electrode position was captured 3–4 times in the total set of images. These images were input into ElectrodeWizzard (von Ellenrieder *et al* 2013) to digitize the electrodes into a three-dimensional space (figures 3(A) and (B)) and incorporated into Matlab Version R2018b (The Mathworks, Inc., Natick, MA, USA). Eight electrodes (9,10, 19, 20, 21, 22, 25, 26) were not included because they lay in a region outside the original infant head model even after scaling to the 4 week old animal. These electrodes cover a region of the head that is behind the ears and toward the neck of the animal and are less likely to capture unique activity related to the brain not captured by other electrodes (see highlighted electrodes inside red polygon in figure 3(C)). Although electrodes covering this area were not included in the model, the remaining 24 electrodes were used to estimate sources of electrical activity projected in five brain regions that include the occipital region (front, left and right temporal, left and right occipital; figure 3(D)).

The 3-D electrode cloud from the remaining 24 electrodes was then co-registered to the piglet model using an affine transformation based on hand-selected fiduciary points and projecting them to the scalp surface using functions from the iso2mesh package (Fang and Boas 2009). The lead field (LF) matrix was then computed using first order FEM with the Galerkin approach, considering the x, y, z, orientation of every brain element as a possible source of activity. We assigned literature conductivity values for each tissue: 0.4 S m^−1^, 0.03 S m^−1^, 1.79 S m^−1^, 0.5 S m^−1^ and 1.5 S m^−1^ for the scalp, skull, cerebral spinal fluid (and ventricles), brain and eyes, respectively (Fernandez-Corazza *et al* 2016).

The inverse solution was estimated using the classical standardized low-resolution brain electromagnetic tomography algorithm (sLORETA) (Pascual-Marqui 2002). sLORETA is one of the most popular inverse solvers due to its algorithmic simplicity and unbiased nature (Sekihara and Nagarajan 2008). The formulation is:

\begin{eqnarray*}{\bf{s}}\left(r\right)=\displaystyle \frac{{\bf{l}}{\left(r\right)}^{T}{\left({\bf{L}}{{\bf{L}}}^{{\bf{T}}}+\alpha {\bf{I}}\right)}^{-1}{\bf{Y}}}{\sqrt{{\bf{l}}{\left(r\right)}^{T}{\left({\bf{L}}{{\bf{L}}}^{{\bf{T}}}+\alpha {\bf{I}}\right)}^{-1}{\bf{l}}\left(r\right)}},\end{eqnarray*}

where **Y** is the *L* × *T* EEG data matrix (where *L* is the number of channels and *T* is the number of time samples), **L** is the LF matrix, **I**(*r*) is the column of the LF matrix generated by the dipolar source at *r*, *α* is the hyperparameter, **I** is the identity matrix and **s**(*r*) is the source waveform estimate of dipole *r*. Formulation in equation (1) is repeated for each possible source of activity. The hyperparameter was selected by visual inspection of a few samples of the source reconstructions and then it remained fixed for the rest of them.

EEG waveform data corresponding to the 24 electrodes that were successfully incorporated were input into the model for source localization. Source localizations were run for each animal per day, per stimulus type (3 days x 3 stimulus types x 11 animals) for a total of 99 source localizations. For each of the source localizations, the mean current density was extracted for five regions of interest and three time points (described below).

### Statistical analyses—peak amplitudes and latencies

To determine if visual, auditory-standard, or auditory-target elicit EP responses in the 4 week old piglet, paired t-tests were used to compare group (*N* = 11) *P*1, *N*1, *P*2, *N*2 amplitudes to zero for each electrode region and stimulus type. To determine the effect of Stimulus Type (3 levels: auditory-standard, auditory-target, and visual), Electrode Region (4 levels: front, temporal left, temporal right, and occipital), and day (1, 2, 3) on EP amplitudes and latencies, 3-Way Mixed Measures ANOVAs with a repeated measure for day was run on each dependent variable separately. For the ANOVA, electrode regions were used and defined as electrodes covering four regions (front, temporal left, temporal right, occipital) where the extracted data from each electrode in the region were averaged to determine a single value representing the regional response (figure 1). Target and standard data were treated as separate levels of the independent variable, Stimulus Type, to see if there was an effect of tone type. Post hoc analyses included one-way ANOVAs and paired t-tests if significant interactions were found. Statistics were conducted using IBM SPSS Statistics for Windows, Version 27.0 (Armonk, NY). Significance was accepted at *p* < 0.05.

### Statistical analyses - source localization

Regional mean current densities were extracted from the brain model for each of the 5 regions at the following timepoints, 50, 85 and 110 ms post stimulus to capture early, peak, and late cortical activity occurring around the *N*1 amplitude. From table 1, the mean *N*1 amplitude for the group tended to have the largest amplitude (uV) for each stimulus type and therefore current densities around this component were selected for source localization analysis. To determine the effect of Stimulus Type (3 levels: auditory-standard, auditory-target, and visual), Brain Region (5 levels: frontal, left temporal, right temporal, left occipital, right occipital), and day (1, 2, 3) on current density, 3-Way Mixed Measures ANOVAs with a repeated measure for day was run at each time point (50 ms, 85 ms and 110 ms). Similar to EP analysis, target and standard data were treated as separate levels of Stimulus Type, to see if there was an effect of tone type. Post hoc analyses included one-way ANOVAs and paired t-tests if significant interactions were found. Statistics were conducted using IBM SPSS Statistics for Windows, Version 27.0 (Armonk, NY). Significance was accepted at *p* < 0.05. To illustrate and qualitatively compare the activation patterns from visual and auditory stimuli, EEG data were taken from a single animal, averaged across the three days studied, and were input into the FEM piglet model to visualize current density maps for different views of the brain for visual and auditory-standard stimuli.

**Table 1.** *P*1, *N*1, *P*2, *N*2 amplitudes and latencies (means and standard deviations) for standard, target, and visual stimuli and each electrode group for all animals (*n* = 11). Amplitudes that are signficantly different from zero (*p* < 0.05) at each electrode Region are in bold text. Occip. = occipital.

	*P*1 amplitude	*N*1 amplitude	*P*2 amplitude	*N*2 amplitude	*P*1 latency	*N*1 latency	*P*2 latency	*N*2 latency
Standard (mean, st dev.)								
Occip.	0.11 (0.59)	**−0.89 (0.78)**	**0.84 (0.73)**	**−0.48 (0.65)**	46.8 (32.4)	91.7 (31.9)	136.1 (28.8)	183.6 (37.6)
Front	**−0.33 (0.54)**	**−2.87 (1.19)**	**1.18 (1.23)**	−0.08 (1.08)	30.8 (14.7)	89.5 (7.4)	162.1 (22.8)	185.1 (49.8)
Left	−0.23 (0.75)	**−2.30 (1.03)**	**0.70 (0.68)**	**−0.83 (0.84)**	39.6 (15.1)	89.0 (14.8)	144.9 (19.3)	181.5 (37.3)
Right	−0.10 (0.60)	**−1.92 (0.95)**	**1.03 (0.81)**	**−0.59 (0.98)**	37.0 (14.8)	87.0 (14.3)	140.7 (22.1)	174.6 (34.6)

Target (mean, st dev.)								

Occip.	**0.37 (0.48)**	**−1.01 (0.87)**	**1.28 (0.92)**	**−0.56 (0.85)**	41.8 (15.8)	81.4 (6.0)	122.5 (18.3)	190.5 (34.5)
Front	0.16 (0.69)	**−2.67 (1.24)**	**1.41 (1.22)**	**−0.42 (1.01)**	37.1 (13.5)	94.0 (7.2)	143.4 (18.8)	190.7 (32.5)
Left	0.21 (0.67)	**−1.73 (0.77)**	**1.42 (0.96)**	**−0.69 (0.75)**	44.4 (15.2)	87.3 (12.8)	135.2 (16.0)	185.9 (23.9)
Right	**0.21 (0.58)**	**−1.75 (0.90)**	**1.39 (1.13)**	**−0.60 (0.91)**	42.4 (16.9)	89.2 (10.0)	136.8 (16.9)	183.3 (28.0)

Visual (mean, st dev.)								

Occip.	**0.37 (0.79)**	**−1.76 (1.20)**	**1.65 (1.37)**	**−0.71 (0.78)**	26.7 (18.7)	62.3 (13.8	122.5 (24.5)	188.3 (40.4)
Front	**0.45 (0.61)**	**−2.77 (1.75)**	**2.49 (2.55)**	**−0.71 (1.71)**	26.4 (11.3)	67.3 (12.8)	127.0 (23.2)	207.2 (45.6)
Left	**0.45 (0.72)**	**−2.97 (1.68)**	**2.62 (2.47)**	**−1.28 (1.71)**	20.8 (8.3)	63.1 (15.4)	118.8 (24.1)	190.1 (34.6)
Right	**1.96 (1.27)**	**−1.78 (1.58)**	**3.10 (2.12)**	0.01 (1.36)	38.8 (9.6)	71.8 (14.2)	145.1 (30.7)	211.4 (62.3)

## Results

**Figure 4.**
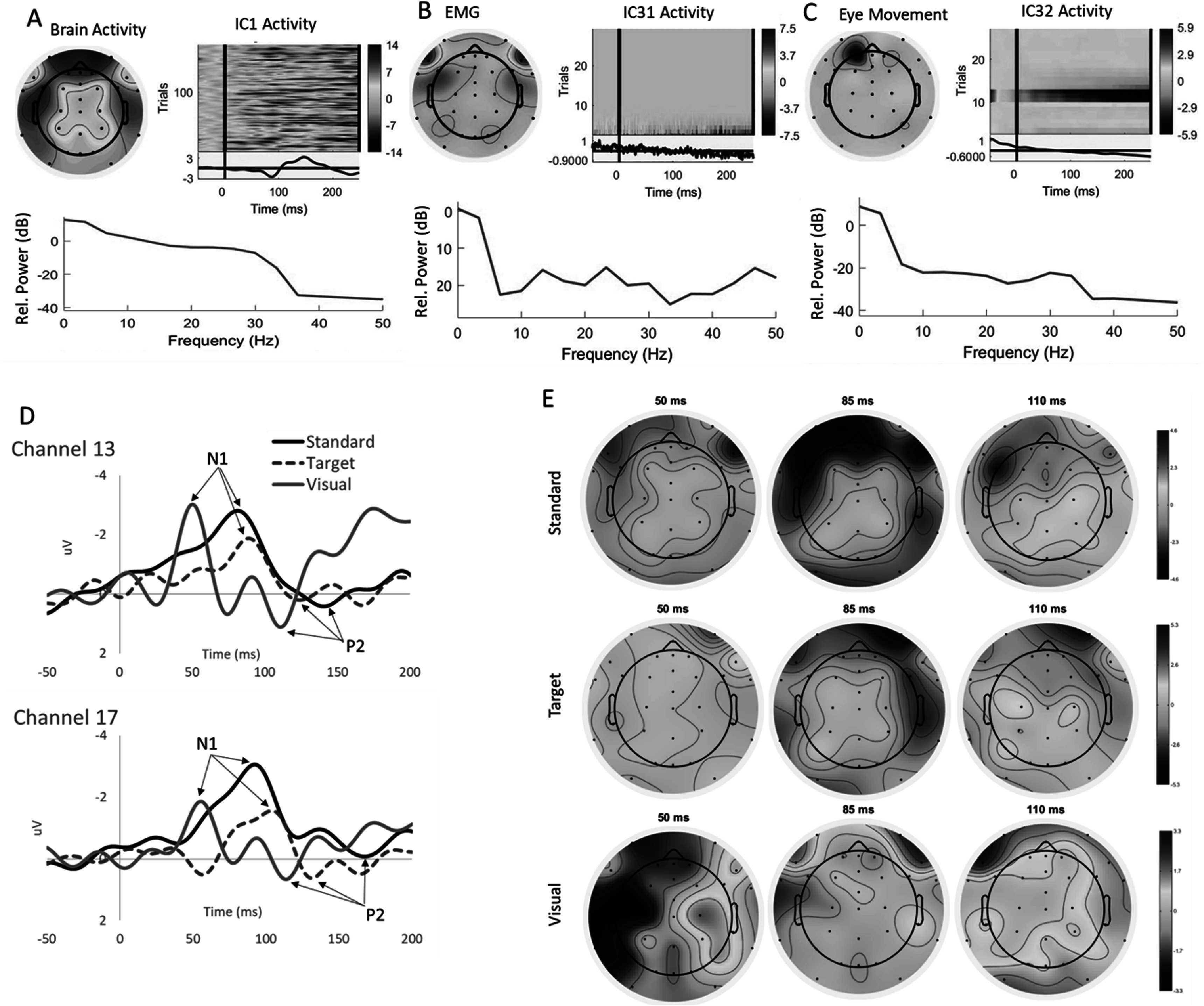
Results from independent component analysis show synchronized brain activity (A), electromyographic activity (B), and eye movement (C). Each panel illustrates the interpolated scalp map, ERP activity color map across all trials with the average ERP (below the color map), and the relative power spectrum (dB) for each event. Waveforms from auditory standard tone (black line), target tone (dotted red line), and visual stimuli (blue line) from channel 13 and 17 with the negative (*N*1) and subsequent positive (*P*2) attributes shown (D). Corresponding topographic maps of the auditory and visual response at 50, 85, and 110 ms post stimuli (E).

Results from the independent component analysis illustrating synchronized brain activity (A) in contrast to spontaneous muscle artefact (B) and eye movement (C) are illustrated in figure 4. Exemplar waveforms from channels 13 and 17 with *N*1 and *P*2 attributes indicated are shown in figure 4(D) along with topographic maps of the auditory-standard, auditory-target, and visual stimuli at 50, 85 and 110 ms shown (figure 4(E)).

### Peak amplitudes and latencies

Table 1 is a summary of *P*1, *N*1, *P*2 and *N*2 amplitudes and latencies (means and standard deviations) and statistical tests evaluating amplitudes against zero for each electrode region are in bold text. For all three types of stimuli, *N*1 and *P*2 amplitudes were consistently different from zero in all four electrode regions. *N*2 amplitudes were significantly different from zero at all four regions for the target tone, however for both standard and visual stimuli, *N*2 was different from zero in only three regions. *P*1 amplitudes however, had a low percentage of values significantly different from zero for standard (one region) and target (two regions) stimuli in comparison to visual (four regions). Overall, *N*1 and *P*2 were the most consistent amplitudes that were different the zero in all three stimuli and all regions.

**Figure 5.**
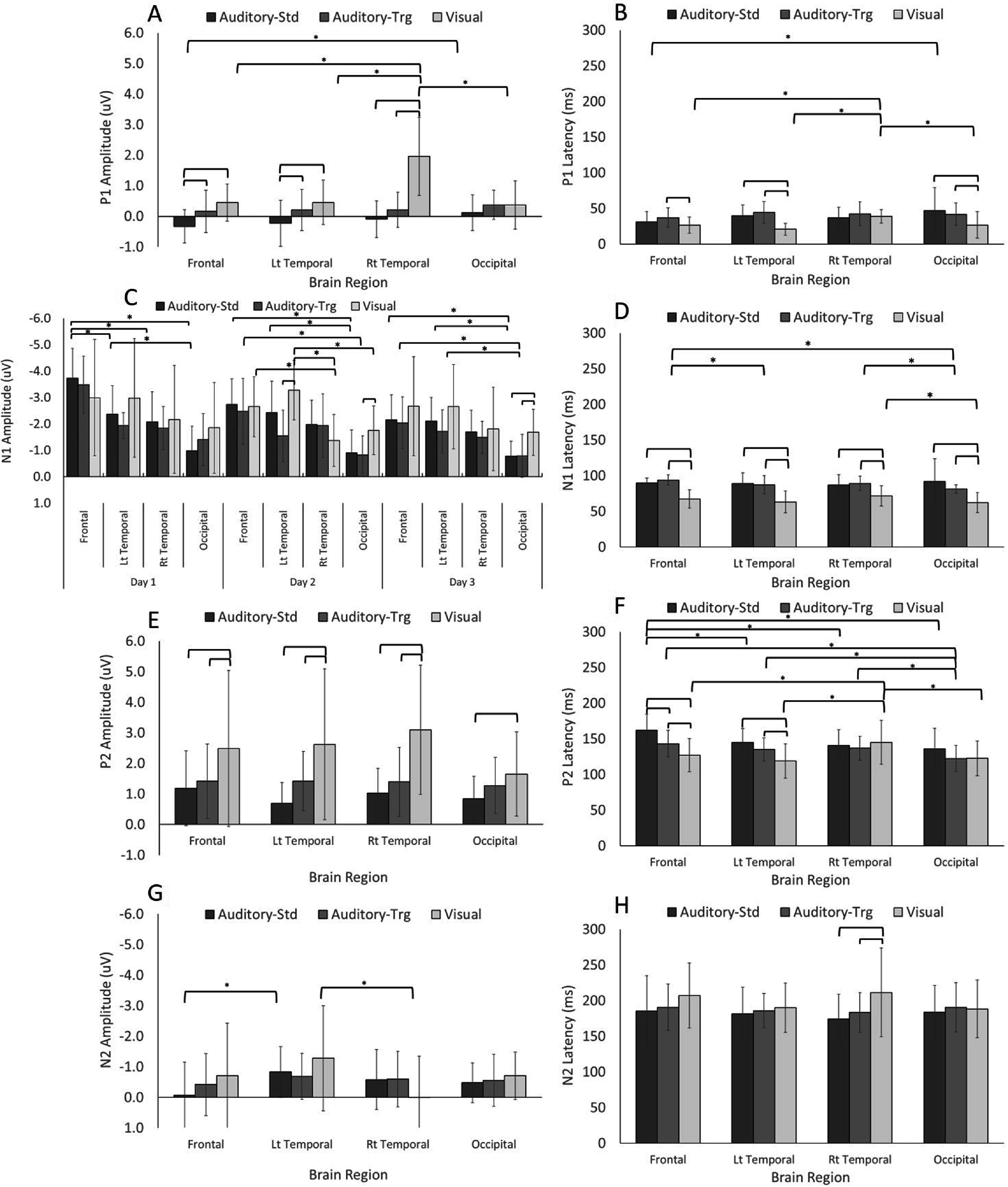
Mean and standard deviations for EP amplitudes and latencies for all animals across brain region and stimulus type (*n* = 11). 2-way interaction effect of brain region and stimulus type on *P*1 (A)–(B), *P*2 (E)–(F), *N*1 (D) and *N*2 (G)–(H) amplitudes and latencies. 3-way interaction effect of brain region, stimulus type, and day on *N*1 amplitude (C). Significant (*p* < 0.05) comparisons **within brain region** are denoted with an overlaying bar. Significant **within stimulus** comparisons are denoted with an overlaying bar and an asterisk. Visual stimuli result in larger *P*1 amplitudes and shorter latencies than auditory (standard and target) stimuli (A)–(B). *N*1 amplitudes were larger and shorter (D) for visual stimuli, particularly at the left temporal and occipital regions reaching significance on day 2 and day 3 (C). *P*2 amplitudes were larger for visual stimuli at all regions (E), and while had shorter latencies for the front and left regions, auditory standard and target had longer latencies for the frontal region in comparison to the occipital (F). Significant *N*2 findings show that left temporal regions had larger amplitudes (G) than the front (standard) and the right temporal (visual) as well as longer latencies for visual stimuli in comparison to auditory at the right temporal region (H).

Table 2 is a summary of the 3-Way Mixed Measures ANOVAs with main and interaction effects identified for each EP and source localization parameter along with the corresponding *F* values, *P* values, and degrees of freedom (DOF). The highest level of interaction effect is further explored and presented. Interaction effects that include the effect with day (region x day and stimulus x day) are not depicted further as subsequent analyses demonstrated that these effects reached significance depending on the day of study and we reason that day effects may be due to the novelty of the stimuli presentation on the first day, resulting in larger activations which are diminished on subsequent days (day 2 and 3). An example of a day effect is shown in figure 5(C) for *N*1 amplitude, where significant relationships between stimulus type and brain region are specific to the day of study.

**Table 2.** Summary of 3-way mixed measures ANOVAs for EP amplitudes and latencies and source localization results at 50, 85 and 110 ms. Significant main and interaction effects between independent variables are reported for each parameter as well the corresponding *F* values and degrees of freedom (DOF).

Variable	Effect	*F* value	*P* value	DOF
*P*1 amplitude	Region	19.065	*P* < 0.001	3.0
	Stimulus	19.089	*P* < 0.001	2.0
	Region x stimulus	18.155	*P* < 0.001	6.0
	Day x region	3.480	*P* = 0.011	6.0
*P*1 latency	Region	4.847	*P* < 0.012	2.0
	Stimulus	12.616	*P* < 0.001	2.0
	Region x stimulus	4.163	*P* = 0.001	6.0
*N*1 amplitude	Region	57.064	*P* < 0.001	2.2
	Day	4.104	*P* = 0.033	1.5
	Region x stimulus	4.819	*P* < 0.001	6.0
	Day x region	4.186	*P* = 0.007	3.6
	Day x region x stimulus	1.841	*P* = 0.012	12.0
*N*1 latency	Stimulus	44.759	*P* < 0.001	2.0
	Day x stimulus	3.362	*P* = 0.015	4.0
	Region x stimulus	2.722	*P* = 0.018	6.0
*P*2 amplitude	Stimulus	7.056	*P* = 0.003	2.0
	Region	5.495	*P* = 0.005	2.2
	Region x stimulus	3.374	*P* = 0.005	6.0
	Region x day	2.574	*P* = 0.037	4.3
*P*2 latency	Stimulus	4.661	*P* = 0.017	4.7
	Region	16.138	*P* < 0.001	2.3
	Stimulus x day	3.281	*P* < 0.017	4.0
	Region x stimulus	7.286	*P* < 0.001	6.0
*N*2 amplitude	Region	7.631	*P* < 0.001	3.0
	Region x stimulus	4.104	*P* < 0.001	6.0
*N*2 latency	Stimulus x day	5.703	*P* < 0.001	4.0
	Region x stimulus	2.452	*P* = 0.031	6.0
Current density—50 ms	Region	12.937	*P* < 0.001	4.0
	Stimulus	23.188	*P* < 0.001	2.0
	Region x stimulus	8.939	*P* < 0.001	8.0
Current density—85 ms	Day	9.650	*P* < 0.001	2.0
	Region	20.041	*P* < 0.001	2.9
	Region x stimulus	5.736	*P* < 0.001	8.0
	Day x region	3.801	*P* = 0.003	4.8
Current density—110 ms	Region	8.714	*P* < 0.001	2.6
	Region x stimulus	4.105	*P* < 0.001	8.0
	Day x region	5.096	*P* < 0.001	4.7

Interaction effects for brain region and stimulus type show that visual stimuli result in larger *P*1 amplitudes and shorter latencies than auditory-standard and -target stimuli (figures 5(A)–(B)) and that visual stimuli are largely detected in the right temporal region. A three-way interaction effect for day, stimulus type, and region revealed focalization of *N*1 response toward the frontal regions for auditory-standard and auditory target, where the *N*1 amplitude was consistently larger at the front than the occipital regions across all three days studied (figure 5(C)). Visual stimuli had larger *N*1 amplitudes at the left temporal and occipital regions than both auditory stimuli, and reached significance on day 2 and day 3 (figure 5(C)). Similar to *P*1, *N*1 latencies were significantly shorter for visual stimuli than both auditory stimuli at all regions (figure 5(D)). A reversal in trends are observed for *P*2 amplitudes, where the visual stimuli had larger responses across regions (figure 5(E)); however, latencies were only shorter at the frontal and left temporal regions than both auditory stimuli (figure 5(F)). Within each stimulus type, the frontal regions tend to have longer *P*2 latencies than most other regions. While *N*2 amplitudes were found to be significantly different than zero in at least 3 out of 4 electrode regions for each stimulus (table 1), the magnitudes of the amplitudes were comparatively smaller than the other parameters and much more variable, despite significant differences found for auditory-standard (left temporal larger than frontal) and visual (left larger than right temporal; figure 5(G)). *N*2 latencies were found to be longer for visual than both auditory stimuli at the right temporal regions (figure 5(H)).

### Source Localization

#### 50 ms

**Figure 6.**
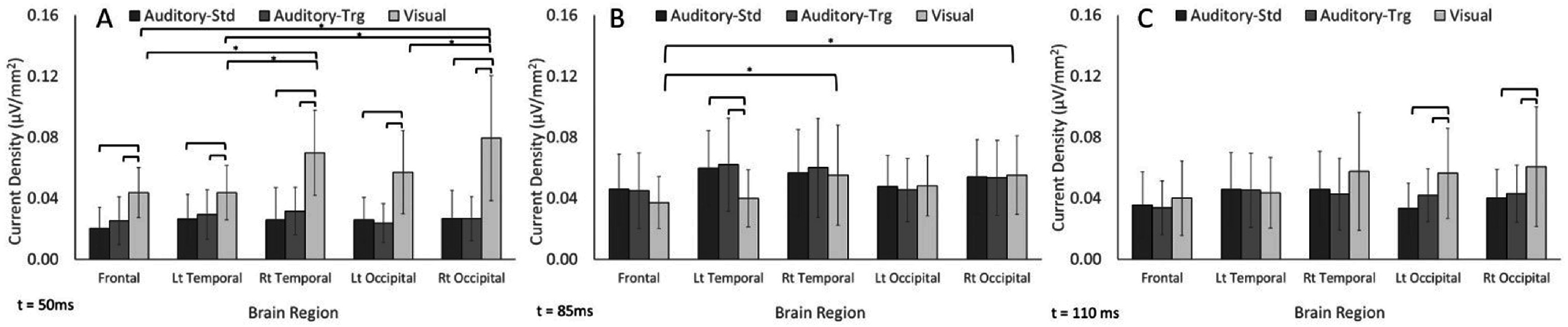
Source localization results demonstrating an interaction effect of brain region and stimulus type on current density at 50 ms (A), 85 ms (B) and 110 ms (C) post-stimuli for 11 animals. Visual stimuli had larger activations at 50 ms, auditory-standard and -target stimuli had greater activations at 85 ms, however at 110 ms, visual had the largest current densities at the left and right occipital regions.

An interaction effect for brain region and stimulus type was found at 50 ms (*p* < 0.05), where visual stimuli had the largest current densities than auditory-standard and -target across all regions. Additionally, a lateralization can be observed at 50 ms, where visual stimuli resulted in significantly greater activity in the right temporal and occipital regions than all other regions (figure 6(A)).

#### 85 ms

At 85 ms post-stimulus, an interaction effect for brain region and stimulus type was similarly observed (*p* < 0.001), however at this time point, the visual stimuli had decreased current densities in comparison to the auditory-standard and -target at the left temporal region (*p* < 0.05). While visual stimuli resulted in lower magnitude current densities at 85 ms than auditory, there was still a localization of activity in the right temporal and occipital regions for visual (figure 6(B)).

#### 110 ms

A brain region and stimulus type interaction at 110 ms post stimuli showed reverse trends than observed at 85 ms where the visual stimuli had larger current densities than both auditory standard and target tones at the right and left occipital regions (figure 6(C)).

**Figure 7.**
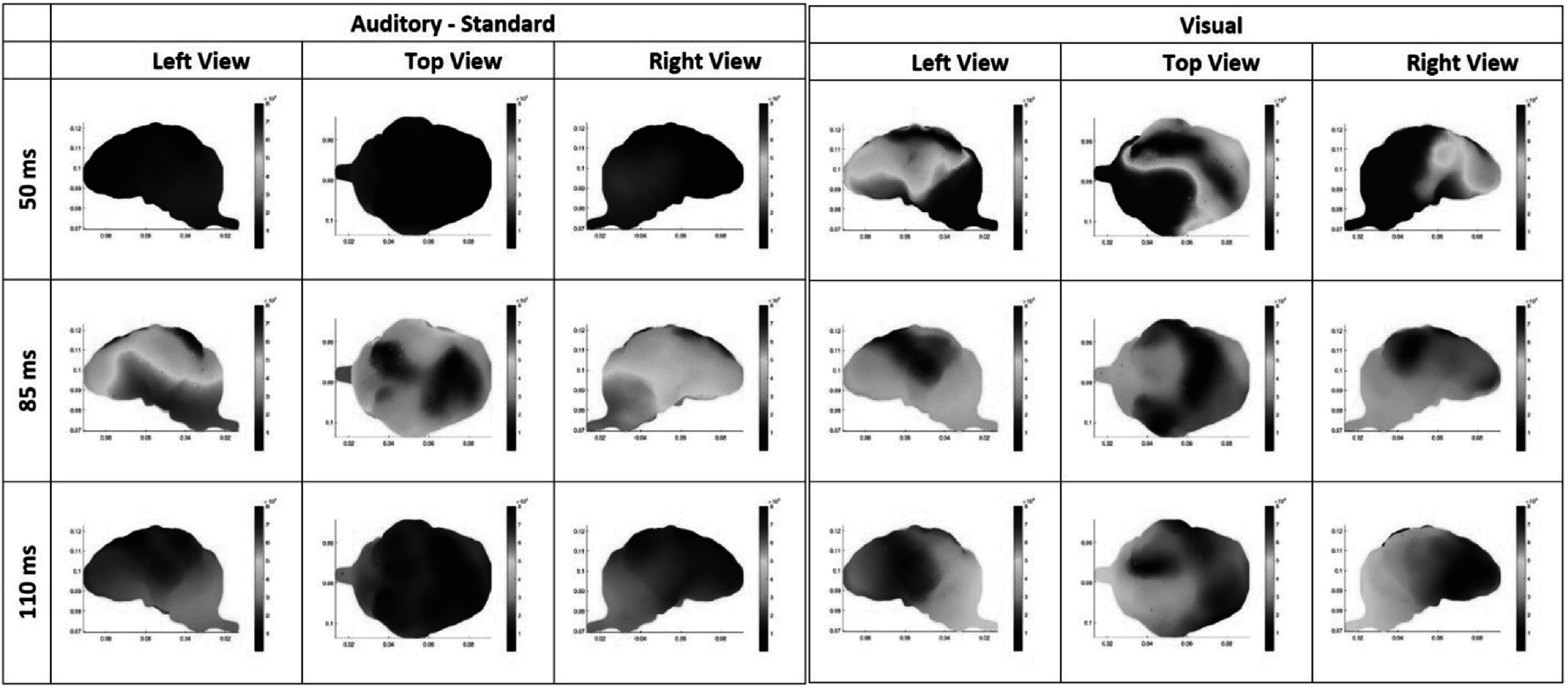
Source localization results showing activation patterns for a single animal where warmer and cooler colors show high and low levels of activations, respectively for auditory-standard (left) and visual stimuli (right) at 50, 85 and 110 ms time points (rows) across a left, top and right view of the brain. The most active brain regions for visual stimuli are the right temporal, left and right occipital regions at 50 ms. For auditory standard stimuli, the greatest activations occur at 85 ms in the frontal and left temporal regions.

Electrical current density maps for visual and auditory-standard stimuli for one exemplar animal is presented in figure 7. The EEG data were averaged across three days to represent the averaged auditory-standard and visual response depicted at 50, 85 and 110 ms. For visual stimulation, maximal cortical activation occurred at 50 ms post stimulus and localized to the temporal and occipital areas as illustrated by the warmer colors. Note that even when excluding the more posterior electrodes due to a model limitation, the remaining electrodes were enough to capture the visual activity in the occipital region. Maximal auditory activation occurred later at 85 ms post stimulus and localized to the frontal and temporal regions. Cortical activation paralleled findings for EPs, where visual stimuli produced *N*1 latencies that were sooner than the auditory-standard tone (figure 5(D)) as well as greater current densities at the earlier time point (50 ms), coinciding with larger *P*1 amplitudes and shorter *P*1 latencies (figures 5(A),(B)). In line with this, auditory-standard tones had longer *N*1 latencies than visual (figure 5(D)) and greater activations at 85 ms (figure 6(B)). Current density maps localized auditory activity to the temporal region and visual activity at the occipital (figure 7).

## Discussion

We demonstrate that visual and auditory stimuli evoke responses in the 4 week old piglet and can be successfully detected and source localized using a 32-channel EEG sensor net and a piglet finite element head model. *N*1 and *P*2 amplitudes were the most consistently measured attributes of the encephalographic waveform for both auditory and visual modalities. However, significant interaction effects found between brain region and stimulus type for both EP and current density demonstrate that auditory and visual stimulation in the 4 week old piglet elicit cognitive responses specific to each modality. Visual stimuli tend to elicit EP responses that are earlier (increased *P*1 amplitudes, decreased *P*1 and decreased *N*1 latencies) than auditory stimuli which coincides with current density results where cortical activations at 50 ms were greater at a number of brain regions for visual in comparison to auditory. At 85 ms, however, auditory stimuli tend to have greater activations than visual, and this can be observed in the larger *N*1 amplitudes for auditory stimuli (figure 5(C)). The shorter EP latencies and earlier current density activations associated with the visual stimuli in our study likely reflects the decreased time required for information processing from a single stimulus presentation in the visual trials in contrast to the two-stimulus (target and standard) auditory oddball tasks which require an element of sound discrimination between the two tones (Polich and Margala 1997, Işoǧlu-Alkaç *et al* 2007).

The visual *P*1, *N*1, *P*2 and *N*2 components in pigs are likely comparable to the human *P*40, *N*70, *P*130, and *N*240 (Allison *et al* 1977). In this study, visual EPs were collected in human subjects who were presented with an un-patterned white light stimulus, similar to the stimuli presented to the pigs in this study. The authors reported ranges of amplitudes and latencies from electrodes from across the head. The *P*40 ranged from 0.4 to 1.9 uV and 38.8 ± 4.7 ms, *N*70 from −1.8 to −7.3 and 70.5 ± 5.7 ms, *P*130 from 0 to 40.2 uV and 128 ± 17 ms, *N*240 from 1.3 to −6.0 and 236 ± 18 ms (Allison *et al* 1977). All pig visual amplitudes and latencies across electrode regions were found to fall within the ranges for the human values presented with the exception of *N*2 latency (table 1). *N*2 latency was faster for pigs (188.3 to 211.4 ms) than in humans (236 ± 18 ms). The range of amplitudes for all components are greater for humans over all, for example, the largest *N*1 amplitude was 40.2 uV, in comparison to pigs (−2.94 uV).

For auditory EPs, comparisons are made with a subset of human data from (Schroder *et al* 2014). In this study, patients with misophonia and control subjects were presented with an auditory oddball sequence along with a silent movie and instructed to ignore the tones. In this way, the presentation of the auditory stimuli were passively processed in a manner consistent with the auditory oddball tasks in our study with pigs, as most studies involving humans requires an active response when the target tone is presented, i.e. a button press (Gosselin *et al* 2006). The *P*1, *N*1 and *P*2 components for the control subjects in the Schroder *et al* (2014) study (Schroder *et al* 2014), were found to be 0.281 uV at 77 ms, 0.290 at 135 ms, and 0.552 at 187 ms, respectively for standard (1000 Hz) tones and −0.080 at 65 ms, −1.614 uV at 133 ms, and 0.352 uV at 246 ms, respectively for target (4000 Hz) tones. In comparisons with the pig data (table 1), standard *P*1 amplitudes are smaller than in humans, however *N*1 and *P*2 are larger in pigs. For target tones, *P*1 and P2 amplitudes are larger in pigs than humans, however the human *N*1 is within range of the pig amplitudes. Interestingly, auditory *P*1, *N*1 and P2 latencies are shorter in pigs than in humans and human latencies for visual EPs (Allison *et al* 1977) occur faster than auditory EPs (Schroder *et al* 2014), which is consistent with the findings in our pigs.

Regions of electrical activity were also specific to stimulus type where visual stimuli had patterns of activity that were localized to the occipital and temporal regions with some frontal activity, however auditory stimuli primarily activated the frontal and temporal regions (figure 7). These findings coincide with the literature where sensory processing and discrimination in humans typically has a centro-parietal topography which has been found for EP topographies (Justen and Herbert 2018) and functional MRI studies (Kiehl *et al* 2001) studying auditory and visual oddball tasks. A frontal distribution of electrical activity was also observed in male Gottingen minipigs when played an auditory oddball paradigm (Arnfred *et al* 2003). The temporal and parietal activity related to auditory processing observed as a result of source localization is in accordance with EP captured at the epidural surface in anesthetized minipigs where a 90 dB sound pressure level click was played in one ear. The auditory stimulation was found to activate the temporal cortex in the brain (Andrews *et al* 1990). Visual processing highlighted from the current density maps, are consistent with the literature demonstrating that visual stimulation using a tri-color (red, blue, green) checker board to the left eye of awake 4 month old female Bama minipigs placed in a functional magnetic resonance imaging scanner activates the visual cortex in the occipital region (Fang *et al* 2006). Right visual cortex activation from the current density maps observed in figure 7, is characteristic of the crossing over of the optic chiasm as left eye visual presentation causes activity in the right visual cortex. Statistical findings illustrate a day and electrode region, and a day and stimulus type interactions for various parameters. Typically, day 1 had greater responses than at day 2 and 3. These decreased amplitudes and current densities after the first day of stimulus presentation are likely due to the animals giving less attention to these stimuli in comparison to day 1, when the stimuli were more novel.

In summary, visual and auditory stimulation elicit unique responses in the healthy piglet brain and could be a diagnostic tool for swine models of human neurological diseases. Preclinical assessments were found to elicit *N*1 and P2 EP responses that were consistent and produce patterns of cortical activations specific to auditory and visual processing. Swine are an important pre-clinical animal model for the human brain due to similar brain development trajectories, neuroanatomical characteristics that are necessary for studying neural, imaging, processing and brain biomechanics associated with TBI and various diseases such as, Alzheimer’s and Parkinson’s. Auditory and visual EPs are important modalities to investigate as sound and light sensitivities activate different CNS pathways in the brain and are practical because they can be monitored non-invasively and presented passively.

Cognitive processing deficits are commonly reported after severe TBI (Nuwer *et al* 2005, Folmer *et al* 2011), however findings at the mild TBI or concussion level are more diverse, with some studies reporting decreased auditory and visual processing (review by Washnik *et al* (2019)) and others reporting an increase in electrical activity after concussion suggestive of an overcompensation mechanism after injury (Ledwidge and Molfese 2016). Variability in the EP findings for mild TBI patients have been attributed to heterogeneity of subjects (age, pre-existing conditions, etc) and the mechanisms of injury that cause trauma to specific areas of the brain (Folmer *et al* 2011). The swine model of TBI permits control over the biomechanical loading factors related to magnitude, frequency, and direction which is invaluable to understanding the subtle effects of mild TBI on cognitive processing and the time course of these effects. Future work in the swine TBI model will be to increase sample sizes and employ a rapid non-impact head rotation (RNR) to administer closed head injuries at levels consistent with sports-related concussion in humans (Cullen *et al* 2016) and track auditory and visual processing deficits in the acute phase of injury.

## Ethical Statement

Emory University’s Institutional Animal Care and Use Committee (IACUC) approved all procedures under PROTO201800149.
